# Characterization of the core microbiome in tobacco leaves during aging

**DOI:** 10.1002/mbo3.984

**Published:** 2020-01-01

**Authors:** Jiaxi Zhou, Lifei Yu, Jian Zhang, Xiaomin Zhang, Yuan Xue, Jing Liu, Xiao Zou

**Affiliations:** ^1^ Department of Ecology Institute of Fungal Resources College of Life Sciences Guizhou University Guiyang China; ^2^ The Key Laboratory of Plant Resource Conservation and Germplasm Innovation in Mountainous Region (Ministry of Education) Collaborative Innovation Center for Mountain Ecology & Agro‐Bioengineering (CICMEAB) Guizhou University Guiyang China; ^3^ Guizhou Tobacco Industry Limited Liability Company Guiyang China; ^4^ Guizhou Tobacco Company Anshun Branch Anshun China; ^5^ Guizhou Tobacco Company Zunyi Branch Zunyi China

**Keywords:** community structure, core microbiome, function prediction, nutritional components, tobacco aging

## Abstract

Microbiome plays an important role during the tobacco aging process which was an indispensable link in the production and processing of cigarettes. However, the structure and functions of microbiome have not been clarified during the tobacco aging process. In this study, 16S rDNA and ITS amplicon sequencing techniques were used to analyze the core microbiome of 15 tobacco samples from five different aging stages. The whole bacterial microbiome was classified into 29 microbial phyla and 132 orders. *Enterobacteriales* (63%), *Pseudomonadales* (16%), *Sphingomonadales* (8%), *Xanthomonadales* (4%), *Burkholderiales* (4%), *Rhizobiales* (3%), and *Bacillales* (2%) comprised the core bacterial microbiome. The whole fungal microbiome was classified into five microbial phyla and 52 orders. Incertae_sedis_*Eurotiomycetes* (27%), *Wallemiales* (25%), *Sporidiobolales* (17%), *Capnodiales* (5%), *Eurotiales* (2%), an unclassified *Ascomycota* (12%), and an unidentified *Eurotiomycetes* (4%) comprised the core fungal microbiome. FAPROTAX function prediction suggested that the core microbiome has a substantial potential for the carbon cycle, nitrate metabolism, aromatic compound degradation, chitinolysis, cellulolysis, and xylanolysis, but simultaneously, the core microbiome is also a source of human pathogens. The dynamics of the bacterial community were primarily determined by the total nitrogen in tobacco leaves during the aging process, while those of the fungal microbiome were primarily determined by total organic carbon. This study indicated that the core microbiome activities may play an important role in regulating the loss of carbon organic compounds and enhancing the secondary metabolites during tobacco leaves aging process.

## Graphical abstract

This study indicated that the core microbiome was involved in the ecological activities of tobacco leaves during the aging process and may play an important role in regulating the loss of carbon and nitrogen organic compounds and enhancing the secondary metabolites of tobacco leaves.

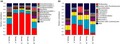

## 1 INTRODUCTION

Flue‐cured tobacco is one of the important cash crops in the world, which requires high quality and security. However, unaged tobacco is unsuitable for cigarette products because it contains strong green miscellaneous gas and produces irritating smoke with an unacceptably harsh flavor (Zhao et al., 2007). In the cigarette industry, a process called natural aging is often used to improve the quality of flue‐cured tobacco. Tobacco aging is a process that changes the physical and chemical characteristics and significantly improves the aroma and flavor of tobacco leaves under certain temperature and humidity conditions. This process typically requires 24 to 30 months (Dixon, Darkis, Wolf, & Hall, 1936), following microbial (or/and enzymatic) actions and other chemical interactions in the leaves (Huang et al., 2010). Microbial activities throughout the aging process are likely to play important roles in improving the tobacco quality during the process.

The bacteria and fungi that inhabit tobacco and tobacco products are varied. The familiar bacterial populations in tobacco include *Bacillus*, *Pseudomonas*, *Sphingomonas*, *Stenotrophomonas*, *Erwinia*, *Pantoea*, *Lactococcus*, *Clostridium*, *Staphylococcus*, *Enterobacter,* and *Paenibacillus (*Chopyk et al., 2017; English, Bell, & Berger, 1967; Huang et al., 2010; Su et al., 2011; Ye et al., 2017; Zhao et al., 2007; Zhu et al., 2001 *)*. The fungal groups include *Aspergillus*, *Penicillium*, *Phoma*, *Alternaria*, *Chaetomium*, *Cladosporium*, *Rhizopus*, *Fusarium*, *Trichoderma*, *Monographella*, *Rhodotorula,* and *Sporidiobolales* (Chen, Wu, et al., 2018; Chen, Li, et al., 2018; Villemur, Lacasse, & Morin, 2009; Welty, 1972; Yang et al., 2008; Zhang et al., 2018). Microorganisms can not only act on the tobacco aging process by their own existence but also through metabolites and enzymes that promote the transformation of biomacromolecules in tobacco leaves, thereby reducing the harmful components, such as nicotine and nitrosamines, increasing the fragrance and improving the quality characteristics (Golias, Dumsday, Stanley, & Pamment, 2000; Liu et al., 2015; Maldonado‐Robledo, Rodriguez‐Bustamante, Sanchez‐Contreras, Rodriguez‐Sanoja, & Sanchez, 2003; Wei et al., 2014).

Tobacco aging is a complex ecological process, and the physical and chemical properties change significantly in tobacco leaves during the process. For example, the total organic acid and volatiles gradually increase, while the nicotine, volatile alkali, total sugar, reducing sugar, and pH values decrease (Sun et al., 2011). After farm curing, tobacco is usually shipped to different countries or regions where it is aged for several months or years, while the storage conditions may vary substantially depending on the location. The water content of tobacco is generally required to be in a controlled range from 10% to 13% during the aging process, but this is usually affected by the humidity of the storage location (Villemur et al., 2009). Moreover, microbial communities in nature are usually complex, and this complexity is exacerbated by interactions among the ecosystem members (Butler & O'Dwyer, 2018). These facts render the analysis of the microbial community structure and function challenging. In addition, the challenge will be further amplified when one considers transient or allochthonous community members, and even though they contribute little to the functionality of the system, they are still detected by the current sensitive molecular approaches (Astudillo‐García et al., 2017). An approach that only considers persistent (and sometimes abundant) members of a microbial community (Shade & Handelsman, 2012) provides a possible way to address these issues.

The core microbiome is considered to be a key component of the basic function of the holobionts, which is enriched, selected, and inherited through evolutionary processes (Lemanceau, Blouin, Muller, & Moënne‐Loccoz, 2017). The core microbiomes are commonly used to reveal microbial species that are closely related to the health, growth, and physiology of the host plant and are currently widely used to describe the key species in human, soil, plant, lake, and wastewater treatment system (Chen, Wu, et al., 2018; Chen, Li, et al., 2018; Ji, Parks, Edwards, & Pruden, 2015; Turnbaugh et al., 2007; Zarraonaindia et al., 2015). However, the core microbiomes and their function in the tobacco aging process still remain to be elucidated. In this study, we used an Illumina Hi‐Seq platform to sequence the 16S rRNA genes and ITS1 amplicons from DNA prepared from 15 tobacco samples that had been stored in three tobacco warehouses of China Tobacco Guizhou Industrial Co., Ltd. We analyzed the core fungal and bacterial microbiomes in tobacco leaves during the aging process. In addition, we also predicted the bacterial functional profiles of the core microbiome in the tobacco leaves using Functional Annotation of Prokaryotic Taxa (FAPROTAX) software.

## 2 MATERIALS AND METHODS

### 2.1 Tobacco storing environment and sampling

The tobacco leaves samples were collected from Baoshan, Yunnan Province, in China. The grade of all the samples was C3F, and the variety of *Nicotiana tabacum* was Yun 87. After harvesting and re‐drying, the tobacco leaves were immediately packed into a carton of 1 m^3^ (density: 200 kg/carton) and transported to Guiyang (GY), Tanchang (TC), and Ziyun (ZY) in Guizhou Province for their respective storage and aging. Each location contained at least three cartons of tobacco, which were stored in a warehouse at ambient conditions where the temperature was between 6°C and 32°C, and the humidity was between 49% and 94%. Sampling began in the first month after all the samples were in storage and continued every six months. A total of 15 samples were obtained with sampling and grouping numbers described respectively as follows: month_0 (GY‐0, TC‐0, ZY‐0), month_6 (GY‐6, TC‐6, ZY‐6), month_12 (GY‐12, TC‐12, ZY‐12), month_18 (GY‐18, TC‐18, ZY‐18), and month_24 (GY‐24, TC‐24, ZY‐24). A five‐point sampling method was used, and five hundred grams of tobacco was sampled at a minimum of 15 cm below the top surface of each tobacco carton (Sun et al., 2011; Villemur et al., 2009). Samples taken from three cartons in the same storage were mixed into one portion with each weighing 1.5 kg. To avoid contamination, sterile gloves and sterile bags were used to sample and seal the tobacco. The tobacco leaves from three locations were sampled within two days and then transported to the laboratory of Guizhou University for storage at −20ºC. In the following experiments, 500 g of each sample was used to determine the chemical components, 500 g was used for microbial analysis, and the remaining samples were used for unavoidable emergencies.

### 2.2 Determination of the nutritional components in tobacco leaves

The nutritional components of the tobacco leaves were determined using the analytical methods of the quality of tobacco chemistry described by Li and Mao (2007). The total organic carbon (TOC) was determined by potassium dichromate–sulfuric acid oxidation at high temperature. The total nitrogen (TN) was determined using concentrated sulfuric acid and the hydrogen peroxide digestion–semi‐microdistillation method. The water‐soluble sugar (WSS) was determined using the arsenomolybdate method. Nicotine was measured using alkaline distillation–ultraviolet spectrophotometry. The starch was determined using the dilute acid–molybdate colorimetric method. The protein was determined using the copper hydroxide precipitation method. The cellulose was treated with acid‐base alcohol ether.

### 2.3 DNA extraction, PCR amplification, and sequencing

The total microorganisms were collected as previously described by Zhao et al. (2007) and Su et al. (2011). The DNA was extracted using the E.Z.N.A.® Soil Kit (Omega Bio‐Tek), and the concentration of DNA and purity were monitored by electrophoresis on 1.0% (*w*/*v*) agarose gels. The primers 515F (5′‐GTGCCAGCMGCCGCGGTAA‐3′) and 806R（5′‐GGACTACHVGGGTWTCTAAT‐3′）were used to amplify the V4 region of the bacterial 16S rRNA gene. The primers ITS5‐1737F (5′‐GGAAGTAAAAGTCGTAACAAGG‐3′) and ITS2‐2043R（5′‐GCTGCGTTCTTCATCGATGC‐3′）were used to amplify the ITS1 fragment of the fungal ITS gene. All the PCR reactions were performed using Phusion® High‐Fidelity PCR Master Mix (New England Biolabs). After quantification, qualification, pooling, and purification, the PCR amplification products were sequenced on an Illumina Hi‐Seq 2,500 platform at Novogene.

### 2.4 Bioinformatics analysis

Raw reads from the original DNA fragments were merged and quality‐filtered using FLASH (Magoč & Salzberg, 2011) and QIIME (Caporaso et al., 2010). The chimeric sequences were subsequently identified and removed using a UCHIME algorithm (Edgar, Haas, Clemente, Quince, & Knight, 2011), and the effective reads were finally obtained. Operational taxonomic units (OTUs) were clustered with a ≥97% similarity cutoff using UParse (Edgar, 2013). The bacterial gene sequences were annotated with taxonomic information using the RDP classifier (Wang, Garrity, Tiedje, & Cole, 2007) against the Greengene database (DeSantis et al., 2006). The OTU taxonomic information of the fungi was obtained by aligning each representative sequence against the Unite ITS database (Kõljalg et al., 2013). OTUs abundance information was normalized using a standard of sequence number corresponding to the sample with the least sequences.

We submitted the OTU biome file to the Metagenomics Core Microbiome Exploration Tool (MetaCoMET), which is a web platform for the discovery and visualization of the core microbiome, and then, we selected the parameters and persistence methods to obtain the core microbiome in tobacco leaves during the aging process (Wang, Xu, Gu, & Coleman‐Derr, 2016; Wang, Lu, Shi, & Xu, 2016). The FAPROTAX was applied to predict the function of the bacterial community in the tobacco leaves. FAPROTAX had been constructed by integrating multiple culturable prokaryotic bacteria whose pronuclear functions had been reported and contained more than 7,600 functional annotations for more than 4,600 species (Louca, Parfrey, & Doebeli, 2016). Furthermore, the relationships between the core microbiome and chemical components were evaluated with CANOCO 5 during the tobacco aging according to the redundancy analysis (RDA).

## 3 RESULTS

### 3.1 Nutritional characterization of the tobacco leaves

**Figure 1:**
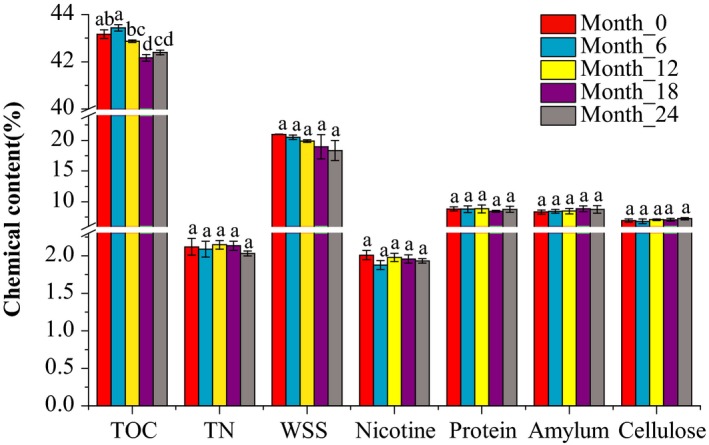
The changes in the chemical components in tobacco leaves during aging. The different small letters show the changes at the .05 level to be significant (*p* < .05)

Carbohydrates and nitrogenous compounds are important components of tobacco leaves and vital sources of nutrition for the tobacco microorganisms. Therefore, we determined the content of the TOC, TN, WSS, nicotine, protein, starch, and cellulose in the tobacco leaves. As shown in Figure 1, the content of the TOC changed significantly during the aging process (*p* = .002) with a decrease from 43.43 ± 0.13% at month_6 to 42.16 ± 0.14% at month_18. The WSS appeared to decrease by 2.63% during the whole process. The contents of the TN and nicotine were relatively low in the tobacco leaves (1.90%–2.33% and 1.69%–2.18%, respectively) and decreased noticeably after six months of aging. There were slight changes in protein, starch, and cellulose, but none were statistically significant.

### 3.2 Bacterial community composition

**Figure 2:**
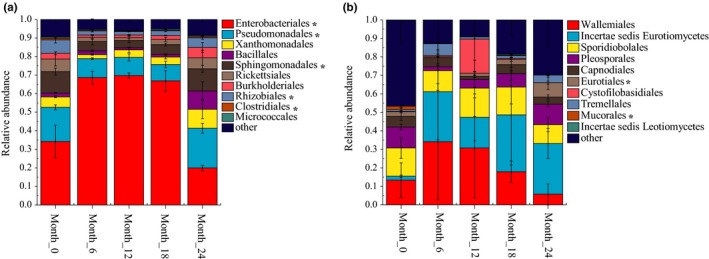
The composition and dynamics of the dominant bacterial (a) and fungal (b) community at the order level. Groups with a relative abundance of <.01 were assigned as “others.” * indicates a significant change (*p* value < .05) in relative abundance of the OTU of tobacco during aging process

**Figure A1:**
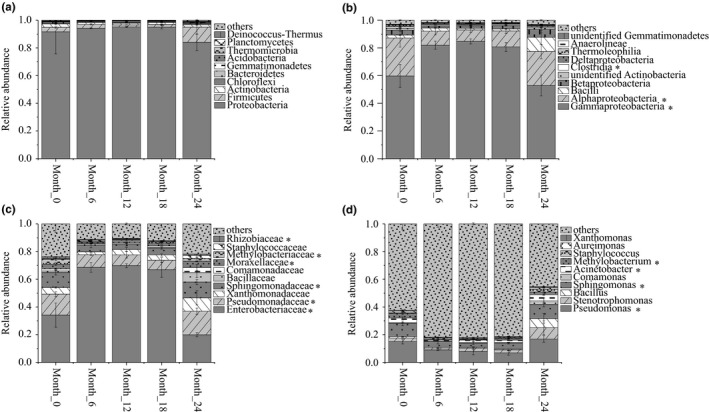
Composition and dynamics of the top 10 bacterial communities at different levels of phyla A, class B, family C and genus D. The relative abundance of other groups with the exception of the top 10 was assigned as “others”. * indicates a significant change (*p* value < .05) in relative abundance of the OTU of tobacco during aging process.

The bacterial 16S rRNA sequencing resulted in 1,242,236 raw reads with 1,191,887 of them passing the quality and length filtering parameters. A total of 2,652 OTUs were obtained by clustering with 97% similarity, including 29 phyla, 66 classes, 134 orders, 264 families, and 490 genera. At the phylum level, *Proteobacteria* had an absolute advantage, accounting for 84.1%–95.2% of the total bacterial communities. In addition, *Firmicutes* and *Actinobacteria* also accounted for 1.5%–10.9% and 1.1%–2.5%, respectively. The relative abundance of *Proteobacteria* decreased from 95.2% to 84.1% after 12 months, while *Firmicutes* increased from 1.5% to 10.9% (Figure A1a in Appendix 1). At the class level, *Gammaproteobacteria* (52.9%–84.8%), *Alphaproteobacteria* (8.3%–27.4%), *Bacilli* (1.2%–10.2%), and *Betaproteobacteria* (1.5%–5.9%) were the primary bacterial groups. The relative abundance of *Gammaproteobacteria*, *Alphaproteobacteria*, and *Clostridia* changed significantly during the aging process (Figure A1b in Appendix 1). At the order level, the bacterial microbiome was dominated by *Enterobacteriales* (19.9%–69.8%), *Pseudomonadales* (8.8%–21.5%), *Sphingomonadales* (3.9%–12.0%), *Xanthomonadales* (2.2%–10.2%), *Rhizobiales* (1.9%–7.2%), *Rickettsiales* (1.6%–6.8%), *Bacillales* (1.1%–9.8%), and *Burkholderiales* (1.4%–5.6%) (Figure 2a). The relative abundance of *Enterobacteriales*, *Pseudomonadales*, *Sphingomonadales*, and *Rhizobiales* changed significantly during the aging process. *Enterobacteriales* increased from 34.2% at the beginning to 69.8% at month_12 and then decreased to 19.9% at month_24. In contrast, the relative abundance of *Pseudomonadales* decreased from 18.4% to 8.8% during the first 18 months and then increased to 21.8% at month_24. During the first 12 months, *Sphingomonadales* and *Rhizobiales* decreased from 11.5% and 7.2% to 4.0% and 1.9%, respectively, but recovered to 12.0% and 5.1% by 24 months. In addition, the relative abundance of *Xanthomonadales* increased from 2.2% at month_6 to 10.2% at month_24. *Bacillales* increased from 1.1% at month_12 to 9.8% at month_24.

In addition, *Enterobacteriaceae* (19.9%–69.8%), *Pseudomonadaceae* (7.0%–17.0%), *Sphingomonadaceae* (3.7%–11.4%), *Xanthomonadaceae* (1.9%–9.7%), *Moraxellaceae* (1.0%–4.5%), *Bacillaceae* (0.6%–6.9%), *Comamonadaceae* (0.4%–3.7%), and *Methylobacteriaceae* (0.6%–3.6%) were the predominant families (Figure A1c in Appendix 1). *Pseudomonas* (6.9%–15.0%), *Sphingomonas* (3.1%–10.1%), *Stenotrophomonas* (0.9%–8.2%), *Acinetobacter* (1.0%–4.4%), *Bacillus* (0.6%–6.7%), and *Methylobacterium* (0.5%–3.4%) were the dominant genera (Figure A1d in Appendix 1). *Enterobacteriaceae* (*Gammaproteobacteria* and *Enterobacteriales*) were the primary members of the *Proteobacteria*, and their relative abundance was significantly reduced after 18 months, while the *Firmicutes* represented by *Bacillaceae* (*Bacilli*, *Bacillales*, *Bacillus*) were significantly increased.

### 3.3 Fungal community composition

**Figure A2:**
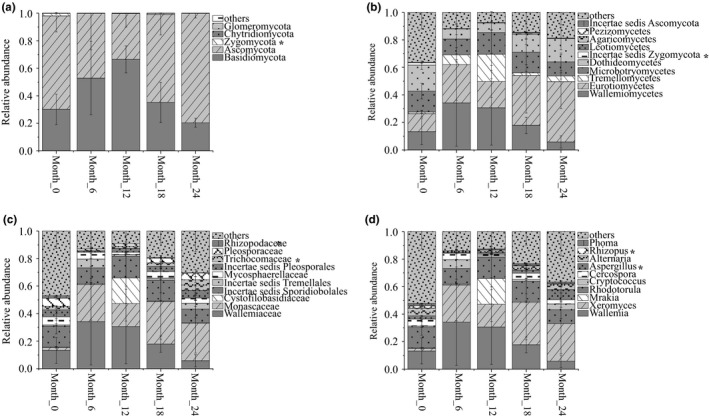
Composition and dynamics of the top 10 fungal communities at different levels of phylum A, class B, family C and genus D. The relative abundance of the other groups with the exception of the top 10 was assigned as “others”. * indicates a significant change (*p* value < .05) in relative abundance of the OTU of tobacco during aging process.

A total of 1,209,369 paired‐end reads were obtained from the 15 tobacco samples using Illumina sequence analysis. After filtering, optimizing, and removing the low‐quality sequences, the remaining 1,173,560 effective sequences were used for the subsequent analyses. A total of 656 OTUs were clustered at 97% similarity and classified into five phyla, 22 classes, 52 orders, 103 families, and 168 genera. *Ascomycota* (33.3%–79.4%) and *Basidiomycota* (20.4%–66.5%) were the dominant fungal phyla at the phylum level. The relative abundance of *Basidiomycota* gradually increased during the first 12 months but decreased gradually later in the process. The Ascomycota fungi reacted in an opposite manner. However, the proportion of other fungal phyla, including *Zygomycota*, *Chytridiomycota,* and *Glomeromycota*, was less than 1% (Figure A2a in Appendix 1). *Eurotiomycetes* (13.0%–43.9%), *Wallemiomycetes* (5.8%–34.2%), *Microbotryomycetes* (10.3%–15.7%), *Dothideomycetes* (6.9%–18.8%), and *Tremellomycetes* (1.2%–19.8%) were the dominant fungal classes (Figure 2b). At the order level, the fungal community was dominated by Incertae_sedis_*Eurotiomycetes* (2.2%–30.8%), *Wallemiales* (5.8%–34.2%), *Sporidiobolales* (10.3%–15.7%), and an unclassified Ascomycota (Figure 2b). In addition, *Pleosporales*, *Cystofilobasidiales*, *Tremellales*, *Capnodiales*, *Eurotiales,* and two unclassified *Ascomycota* also exceeded 1%. The relative abundance of Incertae_sedis_*Eurotiomycetes* rose rapidly from 2.2% to 27.0% in the first six months and declined slightly during the subsequent processing period but exceeded 16%. The relative abundance of *Wallemiales* decreased gradually after 6 months (34.2% to 5.8%). *Sporidiobolales* also showed a gradual decline after 12 months (15.7% to 5.8%).

In addition, *Monascaceae* (2.2%–30.8%), *Wallemiaceae* (13.3%–34.2%), Incertae sedis *Sporidiobolales* (10.3%–15.7%), *Mycosphaerellaceae* (1.7%–5.7%), Incertae sedis *Pleosporales* (0.8%–5.6%), *Pleosporaceae* (1.1%–5.9%), and Incertae sedis *Tremellales* (1.0%–6.4%) were the dominant fungal families (Figure A2c in Appendix 1). Additionally, *Xeromyces* (1.9%–30.7%), *Wallemia* (5.8%–34.1%), *Rhodotorula* (10.1%–15.5%), *Cercospora* (1.1%–5.2%), *Alternaria* (1.1%–5.6%), *Cryptococcus* (1.0%–6.4%), and *Aspergillus* (0.4%–7.4%) were the predominant genera at the genus level (Figure A2d in Appendix 1). *Xeromyces* increased from 1.9% to 27.0% during the first 6 months and was the primary fungal population (abundance over 27%) after 12 months. The relative abundance of *Wallemiaceae* and *Wallemia* decreased from 34.2% and 34.1% to 5.8% and 5.8% after 6 months, respectively, while that of *Trichocomaceae* and *Aspergillus* increased significantly from 0.5% and 0.4% to 7.8% and 7.4%, respectively. *Wallemia* (*Wallemiomycetes*, *Wallemiales*, *Wallemiaceae*), *Rhodotorula* (*Microbotryomycetes*, *Sporidiobolales*, Incertae sedis *Sporidiobolales*), and *Cryptococcus* (*Tremellomycetes*, *Tremellales*, Incertae sedis *Tremellales*) are members of the Basidiomycota. However, their relative abundance gradually decreased during late aging. *Xeromyces* (*Eurotiomycetes*, Incertae sedis *Eurotiomycetes*, *Monascaceae*), *Aspergillus* (*Eurotiomycetes*, *Eurotiales*, *Trichocomaceae*), and *Alternaria* (*Dothideomycetes*, *Pleosporales*, *Pleosporaceae*) are members of the *Ascomycota*, and their relative abundance gradually decreased during late aging.

### 3.4 Determination of the core bacterial microbiome in tobacco leaves during aging

**Figure 3:**
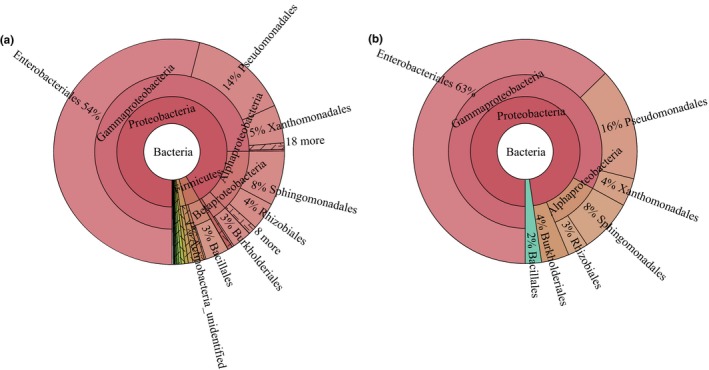
Taxonomic composition of the whole (a) and core (b) bacterial microbiome from tobacco leaves using MetaCoMET. Circles from inside to outside represent the community composition of the bacteria at different classification levels of kingdom, phylum, class, and order, respectively. The size of the fan represents the relative proportion of the annotation results of different bacterial OTU

We used the persistence method to identify the OTUs present across 15 tobacco samples and determine the core bacterial microbiome in tobacco leaves during the aging process. This core bacterial microbiome contained 38 OTUs and corresponded to 83.7% of the whole bacterial microbiome. *Enterobacteriales* was by far the most common and comprised up to 63% of the core bacterial microbiome in tobacco leaves at the order level (Figure 3). However, *Pseudomonadales*, *Sphingomonadales*, *Xanthomonadales*, *Burkholderiales*, *Rhizobiales,* and *Bacillales* also contributed to 16%, 8%, 4%, 4%, 3%, and 2%, respectively. At the genus level (Table 1), an unclassified *Enterobacteriales*, *Pseudomonas*, *Sphingomonas*, *Stenotrophomonas*, *Acinetobacter*, *Methylobacterium,* and an unclassified *Burkholderiales* occupied 63%, 14%, 7%, 4%, 3%, 2%, and 2% of the core bacteria microbiome, respectively. In addition, *Comamonas*, *Bacillus,* and *Staphylococcus* exceeded 1%.

**Table 1:** The dominant bacterial genera (≥1%) within the core bacterial microbiome of tobacco during aging

Number	Taxonomy	Proportion (%)
1	k_Bacteria; p_Proteobacteria; c_Gammaproteobacteria; o_Enterobacteriales; f_Enterobacteriaceae; g_unclassified	63
2	k_Bacteria; p_Proteobacteria; c_Gammaproteobacteria; o_Pseudomonadales; f_Pseudomonadaceae; g_Pseudomonas	14
3	k_Bacteria; p_Proteobacteria; c_Alphaproteobacteria; o_Sphingomonadales; f_Sphingomonadaceae; g_Sphingomonas	7
4	k_Bacteria; p_Proteobacteria; c_Gammaproteobacteria; o_Xanthomonadales; f_Xanthomonadaceae; g_Stenotrophomonas	4
5	k_Bacteria; p_Proteobacteria; c_Gammaproteobacteria; o_Pseudomonadales; f_Moraxellaceae; g_Acinetobacter	3
6	k_Bacteria; p_Proteobacteria; c_Alphaproteobacteria; o_Rhizobiales; f_Methylobacteriaceae; g_Methylobacterium	2
7	k_Bacteria; p_Proteobacteria; c_Betaproteobacteria; o_Burkholderiales; f_Alcaligenaceae; g_unclassified	2
8	k_Bacteria; p_Proteobacteria; c_Betaproteobacteria; o_Burkholderiales; f_Comamonadaceae; g_Comamonas	1
9	k_Bacteria; p_Firmicutes; c_Bacilli; o_Bacillales; f_Bacillaceae; g_Bacillus	1
10	k_Bacteria; p_Firmicutes; c_Bacilli; o_Bacillales; f_Staphylococcaceae; g_Staphylococcus	1

### 3.5 Determination of the core fungal microbiome in tobacco leaves during aging

**Figure 4:**
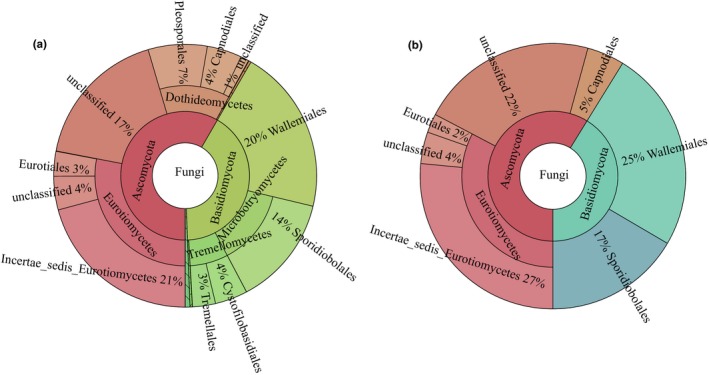
Taxonomic composition of the whole (a) and core (b) fungal microbiome from tobacco leaves using MetaCoMET. Circles from inside to outside represent the community composition of the fungi at different classification levels of kingdom, phylum, class, and order, respectively. The size of the fan represents the relative proportion of annotation results of different fungal OTU

We interpreted the core fungal microbiome for tobacco leaves using the same persistence approach. The core fungal microbiome of the tobacco leaves contained seven OTUs and contributed to 78% of the total fungal microbiome abundance in the tobacco leaves during the aging process. At the order level (Figure 4), Incertae_sedis_*Eurotiomycetes* (27%), *Wallemiales* (25%), *Sporidiobolales* (17%), and an unclassified *Ascomycota* (12%) were the primary dominant fungal orders. *Capnodiales*, *Eurotiales,* and an unidentified *Eurotiomycetes* comprised 5%, 2%, and 4%, respectively. *Xeromyces* (27%), *Wallemia* (25%), a genus of *Ascomycota* (22%), and *Rhodotorula* (17%) were the dominant genera in the core fungal microbiome in the tobacco leaves during the aging process, whereas *Cercospora*, *Aspergillus,* and a genus of *Eurotiomycetes* also comprised 5%, 4%, and 2%, respectively (Table 2).

**Table 2:** The dominant fungal genera (≥1%) within the core fungal microbiome of tobacco during aging

Number	Taxonomy	Proportion (%)
1	k_Fungi; p_Ascomycota; c_Eurotiomycetes; o_Incertae_sedis_Eurotiomycetes; f_Monascaceae; g_Xeromyces	27
2	k_Fungi; p_Basidiomycota; c_Wallemiomycetes; o_Wallemiales; f_Wallemiaceae; g_Wallemia	25
3	k_Fungi; p_Ascomycota; c_unclassified; o_unclassified; f_unclassified; g_unclassified	22
4	k_Fungi; p_Basidiomycota; c_Microbotryomycetes; o_Sporidiobolales; f_Incertae_sedis_Sporidiobolales; g_Rhodotorula	17
5	k_Fungi; p_Ascomycota; c_Dothideomycetes; o_Capnodiales; f_Mycosphaerellaceae; g_Cercospora	5
6	k_Fungi; p_Ascomycota; c_Eurotiomycetes; o_unclassified; f_unclassified; g_unclassified	4
7	k_Fungi; p_Ascomycota; c_Eurotiomycetes; o_Eurotiales; f_Trichocomaceae; g_Aspergillus	2

### 3.6 Functional prediction of the 16S genes using FAPROTAX

**Figure 5:**
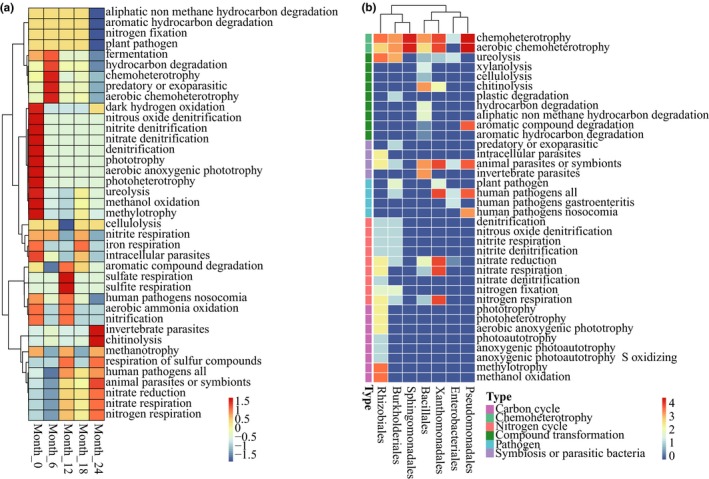
Functional composition of the bacterial community in tobacco leaves using FAPROTAX. (a) The changes of the top predictive gene families. (b) Association of the functional groups with members of the core bacterial microbiome

The 16S sequencing results were selected for additional analysis of their functional prediction because the abundance of bacterial communities obtained from tobacco leaves was far greater than that of the fungal communities. Functional annotation of the OTUs was conducted using FAPROTAX. A total of 68 functional assignments for 703 OTUs (26.5% of the total OTUs) were obtained. Of these, 71.7% and 54.8% OTUs were associated with chemoheterotrophy and aerobic chemoheterotrophy, respectively, indicating that the microorganisms primarily obtained their nutrition by decomposing organic matter in the tobacco leaves. Figure 5a shows that the functional groups, such as denitrification, nitrate denitrification, nitrite denitrification, photoheterotrophy, phototrophy, methanol oxidation, methylotrophy, and methanol oxidation, were the most abundant at the initiation of aging but gradually decreased with the increase in the aging time. Functions, such as hydrocarbon degradation, fermentation, and predation or exoparasitism, were the most abundant at six months, and their proportion was gradually reduced during the subsequent processing. Sulfate respiration and sulfite respiration and aromatic compound degradation accounted for the highest proportion at 12 months. At 24 months, chitinolysis, invertebrate parasites, and animal parasites or symbionts were significant. In addition, the nitrate respiration, nitrate reduction, and nitrogen respiration associated with the nitrogen cycle gradually increased after 12 months, and this change could be related to the accumulation of tobacco‐specific nitrosamines during tobacco aging. In short, with the increase in the aging time, the functional abundance related to carbon metabolism gradually decreased, and nitrate action and parasitism or symbiosis gradually increased after 12 months.

Figure 5b shows the relationship between the core microbiome and the functional group. *Rhizobiales* played an important role in the carbon and nitrogen cycle. *Burkholderiales* promoted nitrogen metabolism and also promoted urea decomposition. *Bacillales* could effectively degrade xylitol, urea, cellulose, chitosan, aliphatic nonmethane hydrocarbons, and aromatic compounds and also participated in nitrate respiration, nitrate reduction, and nitrogen respiration. *Xanthomonadales* had a substantial potential for functions, including nitrate respiration, nitrate reduction, nitrogen respiration, human pathogenicity, and animal parasitism or serving as symbionts. Most *Pseudomonadales* were potential animal parasites or symbionts and human pathogens, but they also played an important role in the degradation of aromatic compounds.

### 3.7 Effects of nutrient substances on the core bacterial and fungal microbiome in tobacco leaves

**Figure 6:**
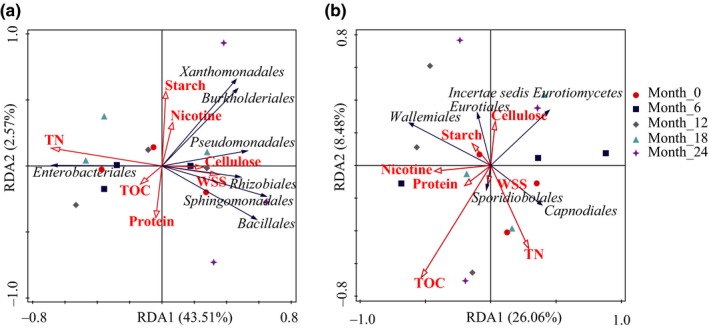
Redundancy analysis (RDA) plot showing the relationship of chemical substances to the core bacterial (a) and fungal (b) microbiomes in the tobacco leaves. TN, total nitrogen; TOC, total organic carbon; WSS, water‐soluble sugars

To further explore the relationship between the nutritional components and microbiome in the tobacco leaves, seven factors, including the TOC, TN, WSS, nicotine, protein, starch, and cellulose, were used in the RDA analysis. As shown in Figure 6, all the chemicals explained 47.3% of the core bacterial microbiome variance and 35.5% of the core fungal microbiome variance, respectively. Among them, the TN influenced most of the core bacterial microbiome and explained 20.5% of the variation in the bacterial composition. The TOC influenced most of the core fungal microbiome and explained 11.4% of the variation of the fungal component. Therefore, we believed that the nutritional component had an important effect on the changes of microbiome in tobacco leaves during the aging process, in which TN and TOC played an important role in the change in the microbiome.

## 4 DISCUSSION

With the rapid development of molecular biology and bioinformatics, the core microbiome has attracted a substantial amount of attention in the fields of microbiology and ecology. Currently, research on the core microbiome has been successively reported. It was found that different ecosystems existed within different core microbiomes, and they played an important role in maintaining the sustainability and stability of the ecosystem. Rui et al. (2015) reported that the core microbiome consisting of *Clostridium*, *Clostridium XI*, *Syntrophomonas*, *Cloacibacillus*, *Sedimentibacter*, and *Turicibacter* could enhance the resistance against environmental stress and maintain digestion efficiency in the household biogas digesters. *Acetobacter*, *Lactobacillus*, *Enhydrobacter*, *Lactococcus*, *Gluconacetobacer*, *Bacillus,* and *Staphylococcus* were functional core microbiota for the production of flavors in Zhenjiang aromatic vinegar (Wang, Xu, et al., 2016; Wang, Lu, et al., 2016). The core bacterial microbiomes were composed of *Gammaproteobacteria*, *Alphaproteobacteria*, *Betaproteobacteria*, *Sphingobacteria*, *Bacilli,* and *Actinobacteria*, and the core fungal microbiome included *Dothideomycetes*, *Leotiomycetes*, and *Tremellomycetes*. They were important components of the plant microbiome and also a gene reservoir of secondary metabolism in Salvia miltiorrhiza seeds (Chen, Wu, et al., 2018; Chen, Li, et al., 2018). This study suggested that the core bacterial microbiome in tobacco leaves during the aging process included *Enterobacteriales*, *Pseudomonadales*, Sphingomonadales, *Xanthomonadales*, *Burkholderiales*, *Rhizobiales,* and *Bacillales* and included Incertae_sedis_*Eurotiomycetes*, *Wallemiales*, *Sporidiobolales*, *Capnodiales*, *Eurotiales*, an unclassified *Ascomycota,* and an unidentified *Eurotiomycetes* form the core fungal microbiome. These core microbiomes play important roles in improving the quality of leaves during tobacco aging.

A large amount of literature showed that microorganisms can reduce the harmful components, regulate the content of chemical components, and increase the aroma substances in tobacco leaf aging. For example, *Pseudomonas* and *Aspergillus* can effectively degrade nicotine. Examples include *P. putida* (Civilini, Domenis, Sebastianutto, & Bertoldi, 1997), *Pseudomonas sp.* Nic22 (Chen et al., 2008), *P. stutzeri* ZCJ (Zhao et al., 2012), *Pseudomonas sp.* HF‐1 (Wang et al., 2013), and *A. oryzae* 11282 (Meng, Lu, Gu, & Xiao, 2010). Wei et al. (2014) found that *Bacillus amyloliquefaciens* DA9 could reduce 32% of the nitrite content and 47% of the tobacco‐specific nitrosamine (TSNA) content in the tobacco leaves during the air‐curing process of burley drying. Maldonado‐Robledo et al. (2003) reported that *Bacillus sp.* could be responsible for the reduction of norisoprenoid to produce 7,8‐dihydro‐ß‐ionone and 7,8‐dihydro‐ß‐ionol. *Burkholderiales*, *Rhizobiales*, *Xanthomonadales*, *Enterobacteriales*, *Pseudomonadales,* and *Sphingomonadales* are important microorganisms in the degradation of furan and/or aromatic compounds in nature (Baraniecki, Aislabie, & Foght, 2002; Wierckx, Koopman, Ruijssenaars, & Winde, 2011). In addition, the *Sporidiobolales* could provide an important pathway for carotenoid synthesis (Frengova & Beshkova, 2009).

Microorganisms could play important roles in the transformation of compounds in tobacco leaves during the aging process. A study by Li (2015) showed that the *Proteobacteria*, *Firmicutes*, *Actinobacteria*, *Bacteroidetes,* and *Basidiomycota* in tobacco leaves before and after storage at different sites were significantly correlated with chemical indicators, including total nitrogen, alkaloids, water‐soluble sugar, and total potassium and chlorine. In this study, the RDA analysis further showed that the changes in the microbial community were closely related to the changes of nutritional components in tobacco leaves (Figure 6). In addition, the FAPROTAX analysis revealed that the microorganisms had the extensive potential to decompose or degrade carbon and nitrogen compounds in tobacco leaves (Figure 5). For example, *Bacillales*, an identified core microbe of tobacco, was isolated the most frequently from tobacco leaves in the aging system, and it could promote the degradation of protein, starch, cellulose, and other compounds, increase the total and reducing sugars, while reducing the amounts of nicotine and total nitrogen (Chen et al., 2016; Li et al., 2015). In addition, three strains of ammonia‐degrading bacteria, including *Stenotrophomonas maltophilia*, *Lysinibacillus fusiformis*, and *Brevibacillus parabrevis*, were also isolated from tobacco leaves and demonstrated a strong ability to degrade organic nitrogen (Zhou et al., 2016). We hypothesized that this process is similar to the degradation of litter in forest ecosystems and that tobacco would be completely decomposed by the microorganisms if given enough time. Therefore, these results indicate that the dominant microbiomes in tobacco aging play key roles in the formation of the quality of tobacco leaves.

However, most fungi were observed in the form of molds and included *Aspergillus*, *Penicillium*, *Alternaria*, *Cladosporium*, and *Chaetomium*. They could be primarily responsible for the postharvest rots and deterioration of tobacco, and they could significantly reduce the quality and use value of tobacco (Welty & Vickroy, 1975). More worryingly, previous studies had shown that some microorganisms (bacteria and fungi) and/or microbial toxins (endotoxins and mycotoxins) in tobacco might lead to the development of diseases, such as chronic inflammation and cancer (Huuskonen et al., 1984; Lander, Jepsen, & Gravesen, 1988; Pauly & Paszkiewicz, 2011; Rooney, Swezey, Wicklow, & McAtee, 2005). Recently, a study of the bacterial metagenomics on four brands of cigarettes showed that there was a broad range of potentially pathogenic organisms in cigarettes, and *Acinetobacter*, *Bacillus*, *Burkholderia*, *Clostridium*, *Klebsiella*, *Pseudomonas,* and *Serratia* were the most notable and accounted for ≥90% of all the cigarette samples. Other pathogenic bacteria detected included *Campylobacter*, *Enterococcus*, *Proteus,* and *Staphylococcus* (Sapkota, Berger, & Vogel, 2010). FAPROTAX analysis in this study found that approximately 7.3% of the microorganisms comprised human pathogenic bacteria in tobacco during the aging process, including *Rhodospirillales*, *Legionellales*, *Pseudomonadales*, *Xanthomonadales* and *Enterobacteriales*. These findings strongly suggest that tobacco or cigarettes themselves could be the direct source of exposure to a substantial amount of potentially pathogenic microbes for smokers and other people exposed to secondhand smoke.

Therefore, the ability of the core microbiome to change has important value for the quality of tobacco aging and the safety of tobacco products. Coordinating the relationship between the beneficial microorganisms and harmful microorganisms will have a significant impact on tobacco producers and smokers in the production and processing of tobacco.

**Figure A3:**
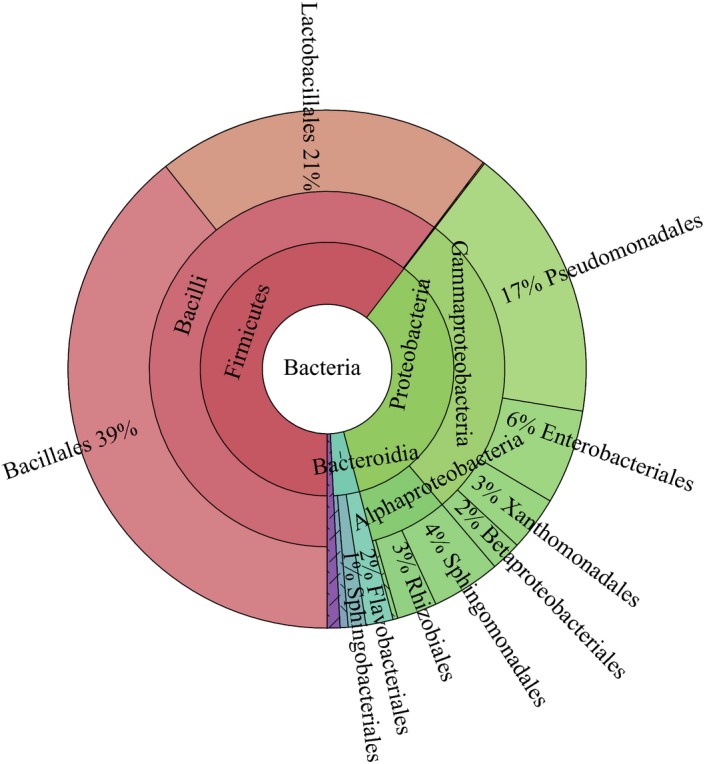
Core bacterial microbiome of tobacco leaves of the variety Zhongyan 100 and grade C3F obtained from Henan Province, China. Data were obtained from the NCBI Sequence Read Archive database (accession number: SRP082278) and had been published by Ye et al. (2017). In our study, we used a total of two tobacco samples for unaged re‐dried (after threshing and redrying, SRR4042109) and aged re‐dried (after 1 year of fermentation, SRR4041956).

**Figure A4:**
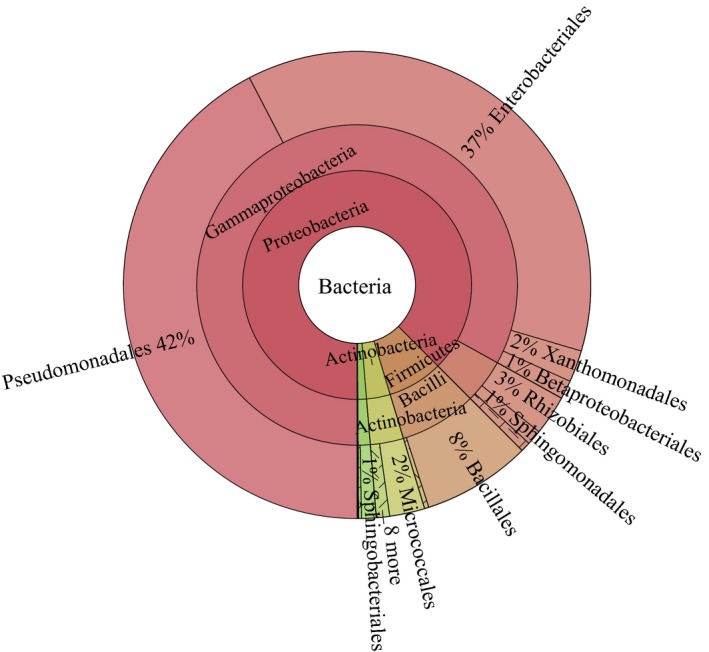
Core bacterial microbiome of the leaves from burley tobacco line TN90H collected from the University of Kentucky's Spindletop Farm. Data were obtained from the NCBI Sequence Read Archive database (accession number: SRP055959) and had been published by Law et al. (2016). In our study, we used a total of 21 tobacco samples, which were air‐cured under conditions of varying heat (15.6–30°C) and relative humidity (60%–90%) in constructed curing chambers.

In addition, *Bacillales* (39%*)*, *Lactobacillales* (21%), *Pseudomonadales* (17%), *Enterobacteriales* (6%), *Sphingomonadales* (4%), *Xanthomonadales* (3), *Rhizobiales* (3%), *Betaproteobacteriales* (2%), *Flavobacteriales* (2%), and *Sphingobacteriales* (1%) are important components of the core bacterial microbiome in tobacco leaves of the variety Zhongyan 100 (Figure A3 in Appendix 1). Additionally, *Pseudomonadales* (42%), *Enterobacteriales* (37%), *Bacillales* (8%), *Rhizobiales* (3%), *Xanthomonadales* (2%), *Micrococcales* (2%), *Betaproteobacteriales* (1%), *Sphingomonadales* (1%), and *Sphingobacteriales* (1%) were the dominant orders of the core bacterial microbiome in the leaves from burley tobacco(Figure A4 in Appendix 1), a type of tobacco, which was air‐cured under various temperature and relative humidity levels. The result of our study was significantly different from those of the study referenced, suggesting that there are differences in the core microbiome of tobacco leaves between different varieties. However, it was found that some microflora, such as *Bacillales*, *Pseudomonadales*, *Enterobacteriales*, *Xanthomonadales*, *Sphingomonadalesetc*, and *Rhizobiales*, seemed to be an important component of the core microbiome of each variety of tobacco, but more evidence is needed to confirm this hypothesis.

## 5 CONCLUSIONS

In summary, microbial diversity is an important component of the tobacco aging ecosystem, and the core microbiome drives the composition and function of the community. In this study, 38 core bacterial OTUs and seven core fungal OTUs were obtained using MetaCoMET software, respectively. The core microbiome has a significant potential application in tobacco carbon metabolism, nitrate and nitrite action, aromatic compound degradation and the decomposition of xylitol, cellulose, and butanol. However, there is a wide range of potentially pathogenic bacteria in tobacco leaves. Therefore, the coordination mechanism between beneficial regulation and the pathogenicity of these microorganisms in tobacco leaves during the aging process merits further study.

## CONFLICT OF INTERESTS

None declared.

## AUTHOR CONTRIBUTION

Jiaxi Zhou: Conceptualization‐Equal, Formal analysis‐Equal, Investigation‐Equal, Writing‐original draft‐Equal, Writing‐review & editing‐Equal; Lifei Yu: Formal analysis‐Equal, Writing‐review & editing‐Equal; Jian Zhang: Investigation‐Equal, Writing‐review & editing‐Equal; Xiaomin Zhang: Investigation‐Equal; Yuan Xue: Resources‐Equal; Jing Liu: Resources‐Equal; Xiao Zou: Conceptualization‐Equal, Formal analysis‐Equal, Investigation‐Equal, Project administration‐Equal, Supervision‐Equal, Writing‐review & editing‐Equal.

## ETHICS STATEMENT

None required.

## Data Availability

All data are provided in full in the results section of this paper apart from the Illumina 16S rRNA, and ITS gene sequences data, which were deposited in the NCBI Sequence Read Archive (SRA) database under the BioProject accession number PRJNA560468, https://www.ncbi.nlm.nih.gov/bioproject/PRJNA560468.
